# Effectiveness and cost-effectiveness of neuromuscular exercise and back care counseling in female healthcare workers with recurrent non-specific low back pain: a blinded four-arm randomized controlled trial

**DOI:** 10.1186/s12889-018-6293-9

**Published:** 2018-12-17

**Authors:** Jaana Helena Suni, Päivi Kolu, Kari Tokola, Jani Raitanen, Marjo Rinne, Annika Taulaniemi, Jari Parkkari, Markku Kankaanpää

**Affiliations:** 1 UKK Institute for Health Promotion Research, Kaupinpuistonkatu 1, 33500 Tampere, Finland; 2 Faculty of Social Sciences (Health Sciences), University of Tampere, Kalevantie 4, 33100 Tampere, Finland; 3 Pirkanmaa Hospital District, Physical and Rehabilitation Medicine Outpatient Clinic, Teiskontie 35, 33520 Tampere, Finland

**Keywords:** Secondary prevention, Early intervention, Exercise therapy, Health education, Costs and cost analysis

## Abstract

**Background:**

Registered healthcare workers worldwide have a high prevalence of work-related musculoskeletal disorders, particularly of the back. Multidisciplinary interventions among these workers have improved fear avoidance beliefs, but not low back pain (LBP) and related sickness absences, cost-effectiveness studies are scarce. Our purpose was to investigate the effectiveness and cost-effectiveness of three intervention-arms (combined neuromuscular exercise and back care counselling or either alone) compared with non-treatment.

**Methods:**

We randomly assigned female healthcare workers with recurrent non-specific LBP to one of four study-arms: Combined neuromuscular exercise and back care counseling; Exercise; Counseling; and no intervention Control. We assessed the effectiveness of the interventions on intensity of LBP, pain interfering with work and fear avoidance beliefs against the Control, and calculated the incremental cost-effectiveness ratios for sickness absence and QALY.

**Results:**

We conducted three sub-studies in consecutive years of 2011, 2012, and 2013 to reach an adequate sample size. All together 219 women were randomized within each sub-study, of whom 74 and 68% had adequate questionnaire data at 6 and 12 months, respectively. No adverse events occurred. Compliance rates varied between intervention-arms. After 12 months, the Combined-arm showed reduced intensity of LBP (*p* = 0.006; effect size 0.70, confidence interval 0.23 to 1.17) and pain interfering with work (*p* = 0.011) compared with the Control-arm. Work-related fear of pain was reduced in both the Combined- (*p* = 0.003) and Exercise-arm (*p* = 0.002). Physical activity-related fear was reduced only in the Exercise-arm (*p* = 0.008). During the study period (0–12 months) mean total costs were lowest in the Combined-arm (€476 vs. €1062–€1992, *p* < 0.001) as were the mean number of sickness absence days (0.15 vs. 2.29–4.17, *p* = 0.025). None of the intervention-arms was cost-effective for sickness absence. There was 85% probability of exercise-arm being cost-effective if willing to pay €3550 for QALY gained.

**Conclusions:**

Exercise once a week for 6 months combined with five sessions of back care counseling after working hours in real-life settings effectively reduced the intensity of LBP, work interference due to LBP, and fear of pain, but was not cost-effective.

**Trial registration:**

ClinicalTrials.gov, NCT01465698 November 7, 2011 (prospective).

**Electronic supplementary material:**

The online version of this article (10.1186/s12889-018-6293-9) contains supplementary material, which is available to authorized users.

## Background

Low back pain (LBP) is among the leading causes worldwide of years lived with disability [1] and has a high economic burden. The annual prevalence of LBP among hospital nurses and nurses’ aids in Europe is between 51 and 57%, and new high-risk groups include home and long-term care nurses and physiotherapists [2]. Many European countries are experiencing a shortage of healthcare workers [3], making it crucial to find ways to reduce the prevalence of long-term LBP and related sickness absence among them.

Physical requirements related to work, such as lifting and transferring patients or working in awkward spine postures [4–6], are major contributors to the high incidence of LBP and injury, and the risk of developing chronic LBP [5]. Among work-related psychosocial risk factors [7], night-shift work [5] and perceived lack of support from superiors [5, 7] are associated with an increased risk of LBP [5, 7] and sick leave in nursing personnel [5]. Fear avoidance beliefs (FABs) [8], a concept explaining how psychologic factors affect an individual’s experience of pain, are prognostic for a poor outcome in subacute LBP [9] and predict sickness absence among healthcare workers [9, 10].

LBP is a condition best understood with reference to the interaction of physical, psychologic, and social influences. In general, patients with subacute LBP who receive multidisciplinary biopsychosocial rehabilitation will do better than if they receive usual care, but it is not clear whether they do better than people who receive some other type of treatment [11]. A recent systematic review on efficacy of interventions for LBP in nurses [12] revealed no strong evidence of efficacy for any intervention in preventing or treating LBP in a nurse population. Post-treatment exercise may reduce LBP recurrence, but the content of an effective program has not been established [13]. Cognitive behavioral interventions, in general, yield improvements in pain, disability, and health-related quality of life [14], but reports of key issues and their operationalization is lacking [15]. Evidence for intense physical conditioning reducing sickness absence in those with subacute back pain is conflicting [16]. High cardiorespiratory and muscular fitness were strongly associated with lower total medical costs in participants of the present clinical trial at baseline [17]. On average, combined physical and psychologic treatments seem relatively cost-effective for subacute LBP [18].

The main purpose of this blind four-arm randomized controlled trial was to study the effectiveness of a 6-month intervention of combined neuromuscular exercise and back care counseling or either intervention alone against a non-treatment control-arm for reducing pain and fear of pain in female healthcare workers with recurrent non-specific LBP. The primary hypothesis was that the combination of neuromuscular exercise and back care counseling would more effectively reduce the intensity of LBP than either intervention alone [19]. In addition, we investigated the cost-effectiveness of combined neuromuscular exercise and back counseling-arm and either alone against the non-intervention control-arm in terms of the incremental cost-effectiveness ratio per reduced days of sickness absence and Quality Adjusted Life Year (QALY) gained.

## Methods

### Study design, settings, and participants

The study-design was a blinded four-arm randomized controlled trial of 6-month interventions with effectiveness and cost-effectiveness evaluations at 12 months. The Ethics Committee of Pirkanmaa Hospital District (ETL code R08157) approved the study protocol (ETL code R08157). The aim of the study, as well as risks and benefits, were clarified in a written information letter to those recruited to the study. Participants were encouraged to continue their usual physical activity and seek any medical or other treatments when needed. All participants provided their written consent to a research secretary at the beginning of the baseline measurements. The study protocol of NURSE-RCT is available at: https://www.ncbi.nlm.nih.gov/pmc/articles/PMC5117067/pdf/bmjsem-2015-000098.pdf [19].

**Fig. 1:**
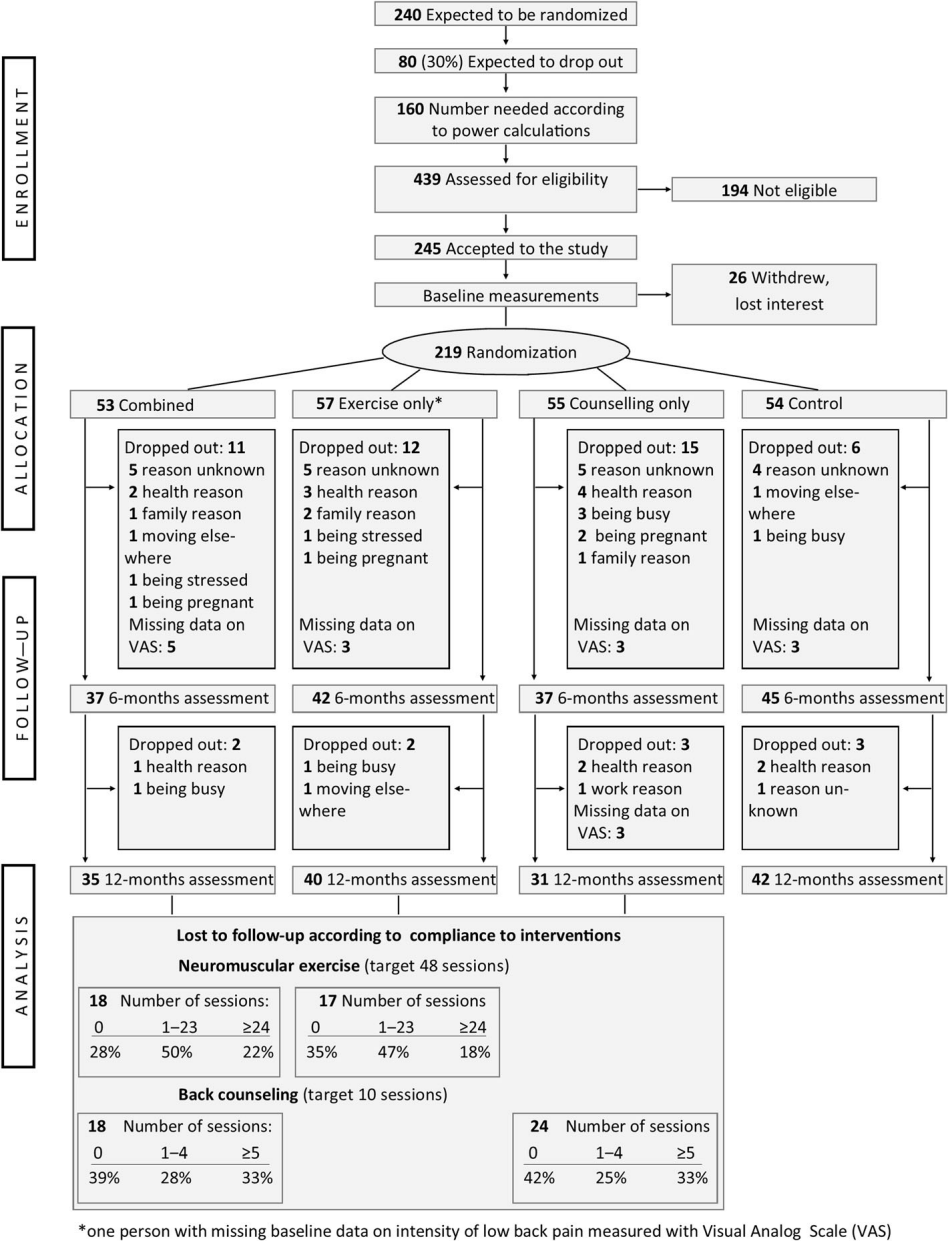
CONSORT flow chart for the main outcome measure (i.e., intensity of low back pain in the past month measured with the Visual Analog Scale) including the number of participants lost to follow-up according to compliance in the tree intervention-arms

Contrary to our original study plan to conduct a single RCT (ClinicalTrials.gov NCT01465698), we conducted three sub-studies to reach an adequate sample size. The sub-studies started consecutively in 2011, 2012, and 2013 at different locations in Tampere, Finland. Details of enrollment, settings, and time-points for screening, randomization, measurements, and interventions for each consecutive sub-study are shown in Fig. 1 of the trial protocol [19].

The target population was female nursing personnel from wards that required lifting and transferring patients, and direct healthcare workers from settings where the work was otherwise awkward for the lower back [6]. In the present paper, ‘healthcare workers’ refers to participants of the present study who were nurses, nurses’ aides, specialist nurses, assistant physiotherapists, physiotherapists, and midwives. The participants were individuals who voluntarily participated in the study on their own time outside working hours.

### Study inclusion and exclusion criteria

The first author (JHS) was responsible for decisions regarding study inclusion or exclusion. The inclusion criteria were [19]: women aged 30–55 years; worked at current job for at least 12 months; intensity of LBP of at least 2 on the Numeric Rating Scale (scale 0–10) during the past 4 weeks [20]. The exclusion criteria were: serious former back injury (fracture, surgery, disc protrusion); chronic LBP defined by a physician or self-report of continuous LBP for 7 months or more [20]; disease or symptoms that limit participation in moderate intensity neuromuscular exercise; regular engagement in neuromuscular-type exercise more than once a week; pregnant or recently delivered. Altogether, 439 women responded to the screening questionnaire, 56% (*n* = 245) of whom met the inclusion criteria and 11% (*n* = 26) of whom refused to participate in the baseline measurements. The main back-related reasons for exclusion were intensity of LBP of less than 2 on the Numeric Rating Scale (22%) and having had continuous LBP for more than 7 months (12%) [17].

### Randomization and masking

Participants were randomly assigned into one of the four study groups in equal proportions within each of the three consecutive sub-studies [19]: Combined neuromuscular exercise and back care counseling (Combined), Exercise alone (Exercise), Counseling alone (Counseling), and a non-treated group (Control). The statistician (KT) generated the random numbers utilizing the RAND function in Excel (Microsoft, Redmond, WA; ver. 2010). At the first appointment, the research secretary obtained a signed informed consent from all participants, after which she opened an envelope (next in order) to allocate the participant to a study group and provide information for participation. Research nurses conducting the study measurements were blind to the group allocation at the time of data collection, and the statisticians (KT, JR) until completion of the statistical analyses.

### Interventions

Educated professionals provided the group exercise and back care counseling sessions near the workplaces of the participating healthcare personnel on weekdays, starting 15 min after the end of a typical day shift [19]. The instructors monitored adverse events related to exercise and adherence to both interventions during the group sessions. Participants in the Combined- and Exercise-arm received instructions to keep a diary of their exercise performed at home.

Researchers AT and JHS were responsible for the aims and training principles of the neuromuscular exercise. AT designed the exercise program and educated the exercise leaders, all of whom had a basic education in physiotherapy, a master’s degree in health sciences, or both. Researchers JHS and MR were responsible for designing the key issues and their realization of the back care counseling sessions. They also guided the counselors, all with a master’s degree in health sciences, to the content and materials of each counseling session at the beginning of each consecutive sub-study.

#### Neuromuscular exercise

The aim of the progressive neuromuscular exercise program (Supplementary appendix 1 of the study protocol: bmjsem-2015-000098supp_appendix1.pdf) was to enhance spinal stability by improving the movement control of the lumbar region of the back [19]. The training principles included maintenance of a neutral spine posture by co-contraction of the trunk muscles in all exercises [21–25]. Exercises demanding high muscular activity and inducing a low lumbar load [24], such as the side-bridge [23] and four-point kneeling [25], were preferred. In addition, exercises to increase the endurance and strength of the gluteal and lower extremity muscles [26] were included to meet the demands of the strenuous lifting tasks required of nursing [18]. The target dose for exercise was 48 sessions (60 min) twice per week for 24 weeks, and the expected minimum efficient dose was 24 based on a previous study by Suni et al. [27]. During the first 8 weeks, the goal was to participate in instructed exercise sessions twice a week, and during the next 16 weeks, in one instructed session and one home session with the help of a digital videodisc or booklet produced for the study [19].

#### Back care counseling

Cognitive behavioral learning theory was the framework for the back care counseling [28], and problem-based learning was the method used for implementation [19]. The main issues introduced and discussed in the group counseling sessions were: explaining LBP; how to avoid harmful loading of the lumbar spine in all daily activities; active strategies to cope with LBP; the role of physical activity in LBP, and overall health and well-being (Supplementary appendix 2 of the study protocol: bmjsem-2015-000098supp_appendix2.pdf). Researchers (JHS, MR) designed the specific learning targets, materials, and “take home tasks” for each session. Safe methods of squatting, emphasizing a neutral spine posture for the lower back [19, 27, 29], were practiced for 5 min during counseling sessions 2–10. The target dose for counseling was 10 sessions (45 min) once a week for the first month and then every third week for 24 weeks.

### Study measurements

The participants took part in study measurements at baseline, immediately after the interventions at 6 months and after follow-up at 12 months. Research nurses conducted the physiologic measurements at the research institute, and the participants responded to the study questionnaire during the measurement sessions or posted them later to the institute.

#### Outcomes of effectiveness

The main outcome measure of effectiveness [19] was intensity of LBP as measured with the Visual Analog Scale (VAS, 0–100 mm) [30]. Secondary outcomes were bodily pain interfering with work [31] and FABs related to work and physical activity [8]. Contrary to the original plan of the NURSE-RCT [19], the results of the test battery regarding movement control impairment [32] are not included in the present paper due to the poor reliability of several test items [33] assessed as part of the first sub-study.

#### Outcomes of cost-effectiveness

We evaluated cost-effectiveness of the three intervention-arms compared with the control-arm in terms of days of sickness absence due to LBP reduced and QALY gained. The QALY were calculated from the SF-6D score [34] derived from the original SF-36 data [31], which is a validated instrument for measuring the physical and mental components of quality of life. Cost assessment included direct healthcare costs (visits to a physician, nurse, physiotherapist, inpatient days, and medication) and days of sickness absence for each 6-month period, retrospectively collected via a questionnaire at baseline [17] and as continuous self-reporting with the same questionnaire during the intervention (0–6 months) and follow-up (6–12 months) periods. We calculated the costs of the delivery of the three intervention-arms: salaries of instructors with administrative costs, material costs, and opportunity cost for home exercise. Additional file 1 provides further information on the SF-6D score [34], assessment of cost-effectiveness and cost calculations, and reports the calculated costs.

### Statistical analysis

#### Sample size calculation

Sample size was calculated [19] based on the intensity of LBP in terms of an absolute change [35] of at least 15 mm in VAS. We expected that there would be a minimal difference of 20% between the intervention groups with improved VAS, and 15% in the control group. Thus, to detect a difference in main effects (i.e., exercisers vs non-exercisers and counseling vs non-counseling) with a significance level of 0.05 and a power of 80%, the study required at least 160 participants (40 in each study-arm). For compensation of probable loss of participants to follow-up, the aim was to recruit 240 participants, see Fig. 1 of the study protocol [19] and the CONSORT flow-chart (Fig. 1) of the present paper.

#### Analysis of effectiveness

The present paper introduces the results of the primary analyses of effectiveness based on a superiority design of any single intervention-arm compared with the Control-arm. Statistician (KT) performed all analyses according to the intention-to-treat principle. The change in the primary outcome of intensity of LBP in VAS and the other outcomes of effectiveness were analyzed as percentage of change [35] over time at three time-points (baseline, 6 months, and 12 months) using a generalized linear mixed model (GLMM) with gamma or log-normal distributions using SPSS statistics software, version 22 (IBM, Chicago, IL).

Statistician KT first conducted the GLMM analyses without any adjustments (crude analysis). Next, the GLMM analyses were first adjusted [36] as follows: *Background variables,* including age, civil status, level of education, and smoking. *Work-related factors* covering shift work, perceived physical exertion at work, perceived work-induced lumbar exertion [37], support from superiors [38], and work stress as effort-reward imbalance [39]. *Health-related factors* included perceived health, perceived fitness, body mass index, meeting the aerobic part of the physical activity recommendation [40], and fitness in a modified push-up test [41]. Only covariates that improved the model at both follow-up stages in the sense of Bayesian information criteria were included in the final models.

Second, the sub-study was included as a random effect in all the GLLM analysis models to indicate possible heterogeneity between the study sites and study time in the three consecutive sub-studies (see Fig. 1 of the study protocol) [19]. KT calculated the continuity-corrected confidence intervals for proportions with the statistical software R function prob.test [42].

We used Cohen’s d to calculate the effect size for the primary outcome measure. The proportion of participants with an improvement of at least 15 mm in the absolute VAS score [30, 35] at 6 and 12 months in each study group is also reported: the between-group differences at the two time-points (baseline and 6 months; baseline and 12 months) were analyzed using the chi-square test.

#### Analysis of cost-effectiveness

We evaluated cost-effectiveness ratio for each intervention-arm in comparison with non-treatment control-arm [43]. Cost-effectiveness is expressed as incremental cost-effectiveness ratios (ICERs), calculated as the ratio of the difference in mean total costs (including LBP related healthcare costs, medication, costs of sickness absence, and intervention costs) and mean effects (i.e., change in number of days of sickness absence or QALY) at the level of the study-arms. Regarding the cost-effectiveness analysis for sickness absence, the costs of sickness absence days of regular workers (i.e. study participants) were not included in order avoid double counting. The ICER indicates the amount of money required to decrease a day of sickness absence or gain QALY.

We estimated the uncertainty regarding the ratios in mean total costs and mean effects using bootstrapping with 5000 iterations to generate 95% confidence ellipses for the joint distribution of cost and effectiveness outcomes, and graphically represented them on a cost-effectiveness plane. Cost-effectiveness acceptability curves indicate the probability of any of the alternative interventions being cost-effective. JR conducted the cost-effectiveness analyses using Stata statistics software, version 12.1 (StataCorp LP, College Station, TX).

The costs of implementing the interventions were higher because we conducted three consecutive sub-studies instead of a single study [19]. To evaluate the robustness of the findings, we performed sensitivity analysis assuming a single intervention for all participants. Thus, the intervention costs related to group-sessions would be one-third of the actual costs.

## Results

### Study participants

All together 219 women were randomized in the three consecutive sub-studies from October 2011 through August 2013 (see Fig. 1 of the study protocol) [19]. Of these 219, 80% (*n* = 176) participated in study measurements at 6 months (intervention period) and 72% (*n* = 157) at 12 months (follow-up period). In the present study, an additional 18 persons were lost to follow-up due to missing data on the main outcome measure (intensity of LBP measured with VAS [30]) as described in the CONSORT flow chart (Fig. 1), which also includes loss to follow-up according to compliance within the three intervention-arms. Almost half of the participants who dropped out did not provide a reason for dropping out; the main reasons for those who did were health-related problems, family reasons, too busy or stressed, and having moved elsewhere.

The background characteristics of the participants are available in Table 1. The mean age of the women was 46 years, mean time in their current job was 11 years, and 70% had shift work. Table 2 provides baseline data on the clinical features of LBP and the study outcome measures. The majority (65%) of the participants reported a pain duration [20] of less than 3 months (i.e., subacute), 40% reported at least a moderate LBP intensity level (≥40 mm in the VAS) [30], and 12% experienced daily pain [20]. Almost a third (31%) of the participants reported multisite musculoskeletal pain of at least moderate intensity (≥4 in numeric rating scale 0–10) at three or more body sites [20]. The majority (78%) of the participants reported no days of sickness absence due to LBP (see Table 3) during the preceding 6 months [17]. The health-related quality of life [34] was in the best third of the highest possible score (Table 2), as was their work ability [44] (Table 1).

**Table 1:** Baseline characteristics of the participants by study groups

Characteristic	Combined (*n* = 53)	Exercise (*n* = 57)	Counseling (*n* = 55)	Control (*n* = 54)	Total (*n* = 219)	Missing (n)
Age (years): mean (sd)	45.1 (6.2)	47.2 (7.4)	46.4 (6.4)	46.7 (7.2)	46.4 (6.8)	0
Years working at current job: mean (sd)	12.1 (9.2)	12.2 (9.3)	9.1 (7.0)	12.4 (9.4)	11.4 (8.8)	2
Civil status: % single	45.3	33.3	32.7	29.6	35.2	0
Education: % secondary school or less	32.1	35.1	49.1	42.6	39.7	0
Shift work: % yes	71.7	64.9	75.9	66.7	69.7	1
Profession:						
% nurses’ aids	37.7	40.4	41.8	42.6	40.6	0
% nurses	56.6	45.6	47.3	37.0	46.6	0
% other	5.7	14.0	10.9	20.4	12.8	0
Work stress, effort-reward imbalance (range 0.2–5): mean (sd)	1.6 (0.5)	1.5 (0.5)	1.7 (0.4)	1.6 (0.5)	1.6 (0.5)	2
Support from superior (range 0–4): mean (sd)	3.3 (0.8)	3.4 (0.7)	3.3 (0.9)	3.6 (0.8)	3.4 (0.8)	1
Work ability index, short form (score 3–27): mean (sd)	21.9 (2.8)	22.0 (2.8)	22.2 (2.8)	22.3 (2.3)	22.1 (2.6)	0
Current smoker: % yes	32.1	19.3	32.7	29.6	28.3	0
Body mass index: mean (sd)	27.1 (5.3)	25.3 (3.9)	26.9 (4.2)	26.4 (4.0)	26.4 (4.4)	3
^a^Meets physical activity recommendation for health: (%)	29.4%	26.4%	20.0%	28.8%	26.2%	13
Muscular fitness: Modified push-ups, reps: mean (sd)	8.9 (3.8)	8.8 (2.9)	9.2 (3.0)	9.2 (2.6)	9.0 (3.1)	6

^a^ objective assessment with accelerometer [45] for 7 days (accepted for analysis if worn minimum 4 days and 10 h/day): aerobic physical activity at least three times per week amounting to at least 150 min of moderate activity or 75 min of vigorous activity (or combination of both), accumulated bouts of at least 10 consecutive minutes

**Table 2:** Baseline data on clinical features of low back pain (LBP) and the outcome measures of effectiveness by study group

Characteristic	Combined (*n* = 53)	Exercise (*n* = 57)	Counseling (*n* = 55)	Control (*n* = 54)	Total (*n* = 219)	Missing
Intensity of LBP; VAS (0–100 mm): mean (sd)	39.9 (20.3)	37.8 (25.7)	32.9 (23.0)	34.5 (20.9)	36.2 (22.6)	1
Proportion with pain intensity of 40 mm or more in VAS: %	47.2	45.5	35.7	31.3	39.9	1
Proportion with daily pain: %	12.8	8.2	17.6	8.9	12.0	27
Duration of symptoms of LBP: %						
(a) < 3 months	64.1	69.1	72.7	51.9	64.5	2
(b) 3–6 months	20.8	12.7	7.3	18.5	14.7	
(c) ≥7 months	15.1	18.2	20.0	29.6	20.7	
Multisite (≥3) musculoskeletal pain with intensity ≥4 on NRS (0–10): %	42.3	29.1	25.5	26.4	30.7	4
Bodily pain interfering with work (SF 36) (score 0–100): mean (sd)	59.3 (17.3)	63.6 (19.8)	65.1 (21.1)	63.6 (17.5)	63.0 (19.0)	8
FABs related to work (score 0–48):^a^ mean (sd)	11.2 (6.9)	11.6 (9.9)	11.0 (7.4)	9.9 (6.9)	10.9 (7.9)	9
FABs related to physical activity (score 0–30): mean (sd)	13.6 (6.6)	14.3 (6.5)	13.8 (6.0)	11.6 (6.0)	13.3 (6.3)	1
Quality of life (SF 36), SF-6D index (0.00–1.00): mean (sd)	0.71 (0.10)	0.74 (0.09)	0.75 (0.11)	0.73 (0.10)	0.74 (0.10)	9
Sickness absence days, previous 6 months: mean (range)	0.8 (0–11)	1.6 (0–40)	1.7 (0–19)	3.4 (0–70)	1.9 (0–70)	16
% with no sickness absence days	79.2	80.0	70.2	81.4	77.6	16
Total healthcare cost^b^ (euros) in previous 6 months: mean (sd)	91 (237)	80 (162)	89 (173)	139 (354)	77 (242)	16
Total costs^c^ (euros) in previous 6 months:mean (sd)	225 (513)	333 (1069)	351 (787)	691 (2582)	400 (1470)	16

*Abbreviations*: *VAS* visual analog scale, *NRS* numeric rating scale, *FABs* Fear Avoidance Beliefs; ^a^questions 10, 15, and 16 excluded as non-relevant in the present study population; ^b^visits to a doctor, a nurse, public health nurse, physiotherapist, in-patient days, medication; ^c^total healthcare costs and costs of sickness absences

**Table 3:** Total costs of low back pain-related direct healthcare costs, intervention costs, days of sickness absence and their costs, and total costs for intervention and total study periods per person in each study group

Characteristic	Combined (*n* = 53)	Exercise (*n* = 57)	Counseling (*n* = 55)	Control (*n* = 54)	*p*-value^a^
Intervention period: 0–6 months					
Total direct healthcare costs: euros (mean; SD)	43 (159)	113 (262)	94 (300)	64 (160)	0.76
Intervention costs: euros (mean)	343	293	46	0	
Sickness absence days: number (mean, range)	0.13 (0–4)	0.86 (0–30)	0.97 (0–16)	1.56 (0–31)	0.60
Sickness absence costs: euros (mean; SD)	48 (244)	315 (1705)	363 (1224)	576 (2020)	0.60
Total costs: euros (mean, SD)	434 (375)	720 (1773)	502 (1457)	640 (2046)	< 0.001
Number of missing cases	14	15	19	13	
Total study period: 0–12 months					
Total direct costs (healthcare costs): euros (mean; SD)	73 (194)	160 (359)	168 (349)	212 (570)	0.28
Intervention costs: euros (mean; SD)	343	293	46	0	
Sickness absence days: number (mean, range)	0.15 (0–4)	4.17 (0–113)	2.30 (0–16)	2.29 (0–51)	0.025
Sickness absence costs: euros (mean; SD)	55 (261)	1529 (7069)	857 (1560)	846 (3212)	0.025
Total costs: euros (mean, SD)	476 (413)	1992 (7317)	1074 (1800)	1062 (3392)	< 0.001
Number of missing cases	19	22	28	16	

^a^Kruskal-Wallis H test

### Compliance with exercise and counseling interventions

We report the compliance of female healthcare workers in the interventions as proportions with a certain number of sessions completed. Exercise sessions (0, 1–23, and 24–48): Combined-arm 9.4, 43.4, and 47.2%; Exercise-arm 10.5, 31.6, and 57.9%. Counseling sessions (0, 1–4, and 5–10): Combined-arm 13.2, 30.2, and 56.6%; Counseling-arm 25.5, 32.7, and 41.8%. No adverse events occurred.

### Effectiveness of interventions

**Fig. 2:**
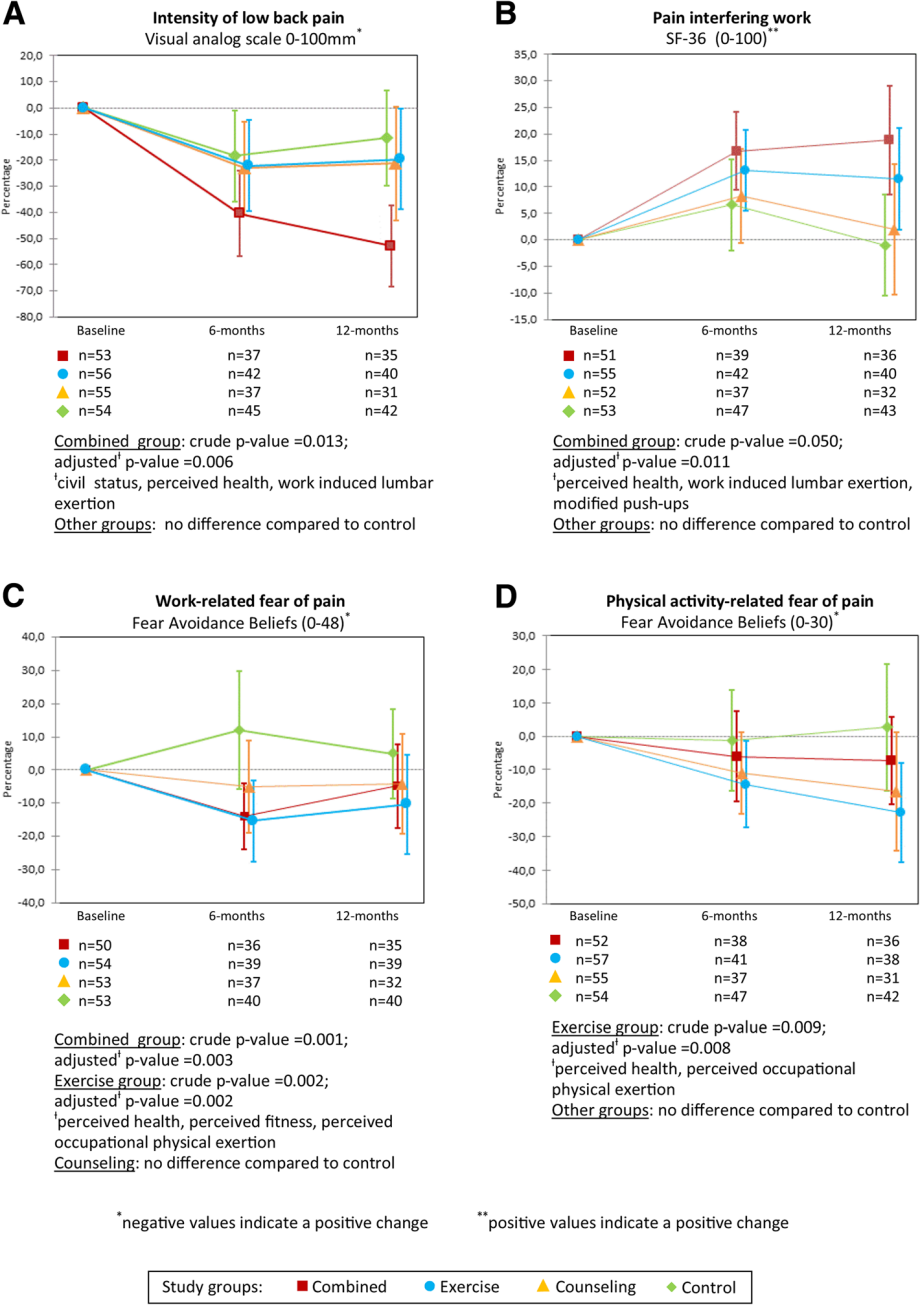
Effectiveness of the four study-arms on (**a**) intensity of low back pain, (**b**) pain interfering with work, (**c**) work-related fear avoidance beliefs, and (**d**) physical activity-related fear avoidance beliefs (mean difference in percentage with 95% confidence interval analysed by general linear mixed models)

The results of the GLMM analysis are available in Fig. 2. The crude *p*-values were somewhat lower compared with adjusted values but differed no more than seven hundredths in any analysis except for the outcome of Pain interfering work (see Fig. 2, panel B), and were almost identical for the two outcomes of FABs (see Fig. 2, panels C and D).

#### Intensity of LBP (primary outcome)

Intensity of LBP (VAS) decreased significantly (*p*-value 0.006) only in the Combined-arm compared with the Control. The effect size (confidence interval) for reduced intensity in the Combined-arm was 0.70 (0.23 to 1.17), the corresponding figures being 0.10 (− 0.37 to 0.57) for the Exercise-arm and 0.09 (− 0.35 to 0.53) for the Counseling-arm.

The proportion of participants with a reduction of at least 15 mm in VAS [30, 35] at 6 and 12 months was as follows: Combined 51.4 and 42.9%; Exercise 40.5 and 25.0%; Counseling 37.8 and 38.7%; and Control 28.9 and 31.0%, respectively. None of the intervention-arms differed significantly (chi-square test) from the Control-arm at any time-point.

#### Other outcomes of effectiveness

Bodily pain interfering with work [31] decreased significantly (*p*-value 0.011) only in the Combined-arm. FABs related to work [8] decreased significantly in both the Combined- (*p*-value 0.003) and Exercise-arms (*p*-value 0.002), and FABs related to physical activity [8] decreased significantly only in Exercise-arm (*p*-value 0.008) compared with the Control (see Fig. 2).

### Costs and cost-effectiveness of interventions

We present the intervention cost, LBP-related use of healthcare services, and days of sickness absence during the intervention (0–6 months) and during the total study period (0–12 months) in Table 3. Costs of sickness absences (*p* = 0.025) and total costs during the total study period (*p* < 0.001) were significantly lower only in the Combined-arm compared with the Control. The sickness absence episodes [17] were mostly short (1–10 days) during both the intervention (85%) and total (81%) study periods. The mean total costs for 0–12 months were as follows: Combined €476, Exercise €1992, Counseling €1074, and Control €1062 (see Table 3).

**Fig. 3:**
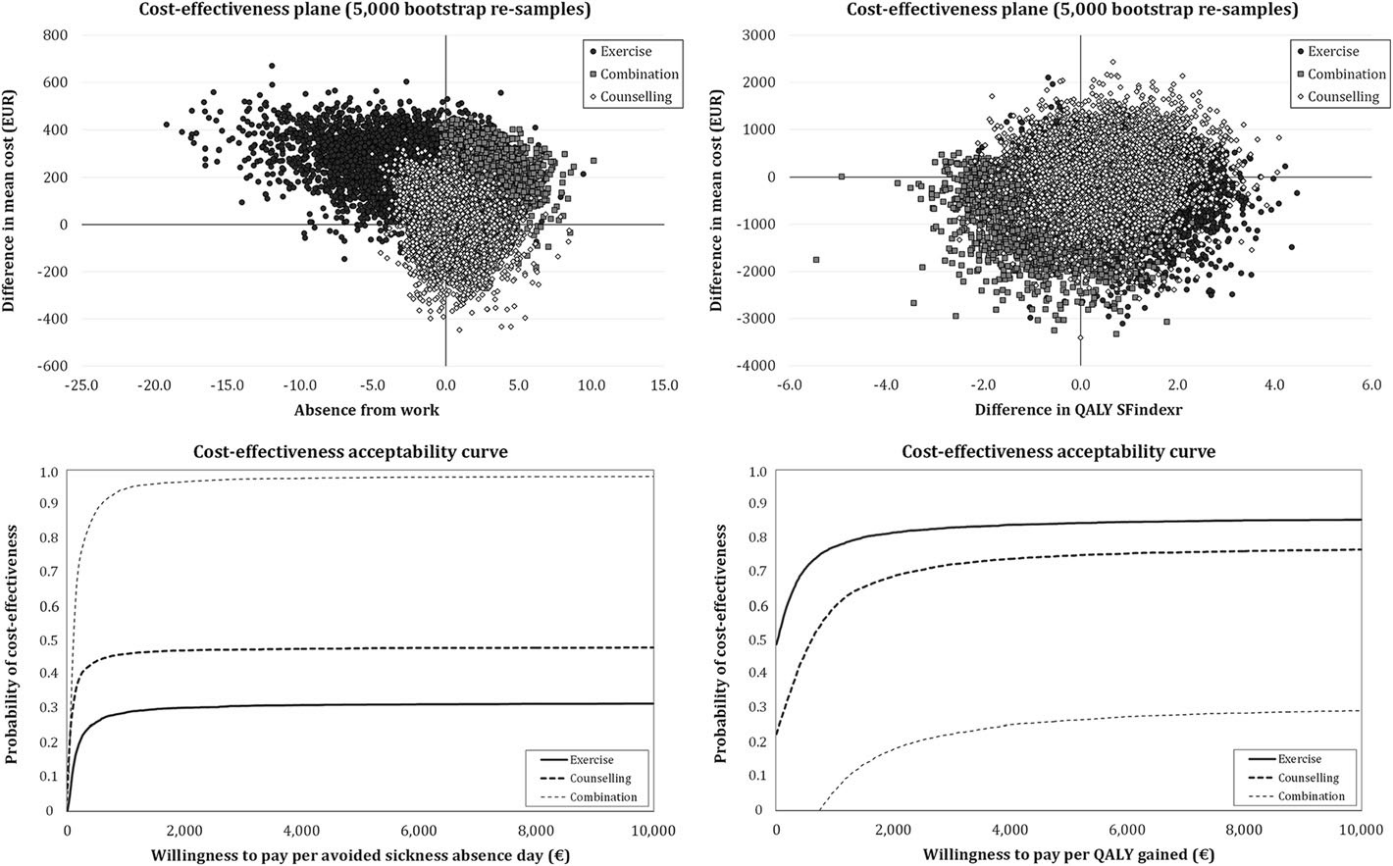
Cost-effectiveness plane and acceptability curve for days of sickness absence from work due to low-back pain, and for Quality Adjusted Life Year (QALY) during the total study period (0–12 months), adjusted for baseline values. ICER indicates the incremental cost-effectiveness ratio

The results of the crude analysis showed that not any of the intervention-arms, when compared with the control-arm, was cost-effective for sickness absence or QALY (see unadjusted results in Additional file 1: Tables S1, S2 and Figure S1). The adjusted results on cost-effectiveness are available in (see Additional file 1: Tables S3 and S4) and Fig. 3. None of the intervention-arms compared with the control-arm was cost-effective for sickness absence after 12-months follow-up (Fig. 3, left panel). There was an 85% probability of the Exercise-arm being cost-effective for QALY at the willingness to pay for €3550 (Fig. 3, right panel). The further sensitivity analyses (i.e. one single study, not three sub-studies) with adjusted variables indicated that none of the intervention arm was cost-effective neither for sickness absence nor for QALY (data not shown).

## Discussion

We assessed the effectiveness and cost-effectiveness of 6-month interventions of combined neuromuscular exercise and back care counseling or either intervention alone compared with no intervention over 12 months in female healthcare workers with recurrent non-specific LBP. In accordance with our hypothesis, the Combined-arm was the only intervention that differed from the non-treatment control-arm regarding pain intensity and interference with work (Fig. 2, panels A and B). Both the Exercise- and Combined-arm were effective for reducing work-related fear of pain, and only the Exercise-arm reduced physical activity-related fear of pain when compared with the control-arm. None of the intervention-arms was cost-effective for sickness absence, the results of Exercise-arm being cost-effective for QALY are uncertain due to the probability of only 85%.

Despite of the fact that none of the intervention-arms was cost-effective in terms of sickness absence, the number of days and costs of sickness absence over the 12 months were significantly lower in the Combined-arm compared with the other intervention-arms (*p* < 0.025, Table 3). As seen in (Additional file 1: Table S3), 3.2% of bootstrap pairs were in the south-east quadrant indicating that the intervention was more effective and less expensive for sickness absence than Control and 95.1% were in north-east quadrant indicating that intervention was more effective and more expensive compared with Control. According to acceptability curve (Fig. 3) there is 95% probability from our data that each avoided sickness absence day requires an additional cost of €1059, i.e. the Combined-arm was not cost-effective due to higher additional costs of each absent day. In Finland, insurance compensates only sickness absences of at least 11 days, thus it is likely that the reduced number of sickness absence days during the 12 months in the Combined-arm translate into substantial savings for the employers. Accordingly, our method of collecting data using a self-report diary for sickness absence seems relevant [17].

We found no previous cost-effectiveness studies of multidisciplinary interventions among healthcare workers. A former intervention including education and light exercise among female hospital workers [46] reduced utilization of painkillers, medical visits, imaging, and outpatient physiotherapy. The present findings on cost-effectiveness slightly disagree with those of a recent systematic review reporting that combined physical and psychological treatments or interventions for LBP are likely cost-effective [18]. The evidence for the cost-effectiveness of physical exercise programs for LBP is inconsistent [18].

A previous study on LBP, FABs, and sickness absence in healthcare personnel [46] reported that although FABs reduced (i.e. improved), there was no effect on LBP recurrence. Another multifaceted (physical training, cognitive behavioral training, and ergonomics) cluster-randomized controlled trial with 594 nurses’ aides [47] also reported reduced FABs, but no effects on sickness absence due to LBP. A recent systematic review on the efficacy of interventions for LBP in nurses [11] demonstrated no strong evidence for the efficacy of any intervention in preventing or treating LBP in populations of nursing personnel. Thus, our findings are more positive for the intensity and interference of LBP compared with these previous studies and the review.

The rationale behind the proposed neuromuscular exercise and back care counseling programs [19] relies on the experiences and findings of two previous studies with male subjects [27, 29]. The common feature of the three studies is that the majority of participants were engaged in physical work that was strenuous for the lower back. In addition, improved movement control of the lumbar spine and awareness of harmful loading for the back “24 h/7d” was an important target. In the present study, we emphasized the movement control of the lumbar back in all neuromuscular exercises during the instructed sessions. During counseling sessions 2–10, the women rehearsed lumbar movement control in terms of the different squat patterns needed in daily life [19].

While LBP reduced only in the Combined-arm, it is possible that the women in this group were the only ones who learned the movement patterns that helped to avoid re-injury. This theoretically relates to the hypothesis that chronic back pain originates from sub-failure injuries of spinal ligamentous and fascial structures leading to muscle fatigue, further injuries, and inflammation [48]. Back pain is a complex multifactorial problem, and therefore a single hypothesis cannot explain all the biopsychosocial factors included in the present study.

Limitations of the study include the lower than expected compliance rate in all intervention groups. The majority of women had shift work, which is likely a challenge that negatively influenced the compliance rates. The dropout rate of slightly over 30% at 12 months is another limitation of the study, but we ended up with an adequate number of participants needed according to our power calculations [19] to ensure adequate statistical power. Of the studies reviewed, the one with the lowest dropout rate of around 12% [46] allowed the nurses to take part in the interventions during working hours, which was not a possibility in the present study. High baseline status may explain the lack of clear cost-effectiveness in improving QALY; a longer follow-up might also be necessary to see changes in outcomes such as QALY.

Study strengths include the four-arm study design, fair adjustment of background, work-related and health-related factors with relevance to our study-group and outcome measures [49], and the success of the Combined-arm in decreasing LBP intensity and interference. On average, neuromuscular exercise no more than once a week during 6 months combined with five sessions of back care counseling was the dose leading to important clinical improvements. We suggest that this dose would be a feasible worksite intervention during working hours, with likely improvements in participation and compliance.

Our decision to recruit women with subacute or recurrent LBP, the majority of whom reported no daily pain (Table 2), offered a real opportunity for the prevention of chronic LBP and sickness absence due to LBP, and thus a future possibility of reducing the socioeconomic burden of LBP in healthcare workers. However, it was extremely difficult to recruit participants that met the inclusion criteria in terms of “non-chronic.” Therefore, we conducted three sub-studies but still the total number of participants at baseline was only 219 compared with the targeted 240, and this is a limitation of the study. Recent studies indicate that structural changes in lumbar muscles in non-specific LBP and FABs differ between patients with recurrent and continuous chronic pain [9, 50]. Thus, our compliance to the pre-determined inclusion and exclusion criteria is another strength of this study.

## Conclusions

The findings of the present study on cost-effectiveness were negative for sickness absence and uncertain for QALY. Contrary to that, the results on effectiveness are encouraging when compared with the rather negative findings of a recently published systematic review and the most recent randomized trials on effectiveness of multidisciplinary interventions aimed at reducing LBP pain among nursing personnel. Neuromuscular exercise no more often than once a week for 24 weeks combined with five sessions of back care counseling is a feasible and effective program for reducing LBP in female healthcare workers. These workers are at high risk for chronic LBP and increased sickness absence due to their physically strenuous work for the lower back.

## Additional file

Additional file 1: Methods Assessment of cost-effectiveness: outcomes measures and cost calculations; Results The calculated costs, **Tables S1–S4.** and **Figure S1.** presenting unadjusted cost-effectiveness plane and acceptability curves for sickness absence from work and for QALY. (DOCX 1439 kb)

## Abbreviations

FABs: Fear avoidance beliefs
GLMM: Generalized linear mixed model
ICERs: Incremental cost-effectiveness ratios
LBP: Low back pain
QALY: Quality Adjusted Life Years
VAS: Visual Analog Scale
