# Supplementary material for: Effectiveness and cost-effectiveness of neuromuscular exercise and back care counseling in female healthcare workers with recurrent non-specific low back pain: a blinded four-arm randomized controlled trial
Source: BMC Public Health. 2018 Dec 17;18:1376. doi: 10.1186/s12889-018-6293-9 (PMC6296156; doi:10.1186/s12889-018-6293-9)
Supplement: Supplementary file 1 — Methods Assessment of cost-effectiveness: outcomes measures and cost calculations; Results The calculated costs, Tables S1–S4. and Figure S1. presenting unadjusted cost-effectiveness plane and acceptability curves for sickness absence from work and for QALY. (DOCX 1439 kb) [file 12889_2018_6293_MOESM1_ESM.docx]

**Additional file 1**

**Contents:**

page

e1. METHODS 1

e1.1. Assessment of cost-effectiveness:

*e1.1.1. Outcome measurements*

*e1.1.2. Cost calculations*

e2. RESULTS 2

e2.1 Detailed unadjusted results of the calculated costs (eTables 1-2) 2

eTable 1. Mean total costs and unadjusted absence from work per person at 1-year follow-up

(6-month intervention and 6-month follow-up) and incremental cost-effectiveness ratio (ICER) 2

eTable 2. Mean total costs and unadjusted QALY per person at 1-year follow-up (6-month intervention and

6-month follow-up) and incremental cost-effectiveness ratio (ICER) 2

eFigure1. Unadjusted cost-effectiveness plane and acceptability curves for absence from work and for QALY 3

e2.2 Detailed adjusted results of the calculated costs (eTables 3-4) 4

eTable 3. Mean total costs and adjusted absence from work per person at 1-year follow-up

(6-month intervention and 6-month follow-up) and incremental cost-effectiveness ratio (ICER) 4

eTable . Mean total costs and adjusted QALY per person at 1-year follow-up (6-month intervention and

6-month follow-up) and incremental cost-effectiveness ratio (ICER) 4

eReferences 5

**e1. METHODS**

**e1.1. Assessment of cost-effectiveness**

*e1.1.1. Outcome measurements*

Cost-effectiveness of the interventions was evaluated in terms of sickness absence days due to low back pain and quality-adjusted life years (QALY). The data were collected at baseline, at the end of the 6-month intervention period and after follow-up at 12 months. Sickness absence (self-report diary) [1] was calculated by adding each day absent from work due to low back pain. We collected the health-related quality of life (HRQoL) data via questionnaires [2, 3].

The QALY were calculated from the SF-6D score [4-6] derived from the original SF-36 data, which is a validated instrument for measuring the physical and mental components of quality of life [3]. The index score covers eight dimensions of HRQoL: physical functioning, physical role limitation, bodily pain, general health, vitality, social functioning, emotional role limitation, and mental health. The index score is expressed by a single value, from 0 (the worst health state) to 1 (the best health state). The QALY gained were calculated for the intervention period (0-6 months) and for 0−12 months. The difference in the SF-6D index for the total study time was calculated by subtracting the baseline score from the 12-month score. The SF-6D index was multiplied by the expected lifetime and calculated for each subject separately.

*e1.1.2. Cost calculations*

The economic evaluation included the healthcare costs to the municipality, costs incurred by the patient, and medication and productivity costs due to low back pain. The information on the use of health care services (i.e., visits to a doctor, a nurse, public health nurse, or physiotherapist and in-patient days), number of sickness absence days, and medication during the last 12 months were taken into account in the analysis. The health care costs were based on average national costs for health care [7]. The unit costs of visits to physicians and to nurses included salary costs and administrative costs, but not laboratory expenses. In addition, the unit cost of inpatient hospital days included the daily inpatient-care charge [7]. Medication costs were beased on mean costs of the pill in pharmacist's. The opportunity cost, which in economics denotes the value of the best alternative forgone, was estimated. Related to intervention costs, the opportunity cost of lost leisure time used for home exercise was taken into account. The travel expenses and time costs related to the use of health services were not taken into account because this information was not available.

All costs, collected with the self-report diary were calculated for the 6- and 12-month periods as continuous self-reporting during the intervention (0-6 months) and follow-up (6-12 months) periods. The unit costs, productivity costs, and medication costs were entered at the price level of each sub-study i.e.Sub-study1 (2012), Sub-study2 (2013), and Sub-study3 (2014) in euros [8]. Intervention costs of all three sub-studies were taken into account. The salary costs were calculated using the friction cost approach from the average national monthly salary scales of practical nurses and of registered nurses according to year of the sub-study [7] multiplied by 1.3 to encompass related expenses [7] and to include the cost of the substitute for the absent nurse. The cost calculation assumed 220 workdays per year. Based on that, the price for one sickness absence day of a nurse was on average €371 [9].

**e2. RESULTS**

**e2.1. Unadjusted cost-effectiveness/utility outcomes: Tables e1-e2**

**eTable 1. Mean total costs and unadjusted absence from work per person at 1-year follow-up (6-month intervention and 6-month follow-up) and incremental cost-effectiveness ratio (ICER)**

|  | | | | **Compared with control group** | | | | | | | | | | |
| --- | --- | --- | --- | --- | --- | --- | --- | --- | --- | --- | --- | --- | --- | --- |
| **Intervention groups** | **Sample size** | **Cost / person (C)** | **Absence from work/ person (E)** | **Incremental cost (ΔC)** | **Incremental effect (ΔE)** | **ICER**  **(ΔC / ΔE)** | **NE** | **SE** | **SW** | **NW** | **85% ICER** | **90% ICER** | **95% ICER** |  |
| Control | 38 | 216 € | 2.29 |  |  |  |  |  |  |  |  |  |  |  |
| Combination | 34 | 421 € | 0.15 | 205 € | 2.14 | 95.8 | 95.8 | 3.2 | 0.02 | 1.0 | 332 € | 465 € | 867 € |  |
| Exercise | 35 | 463 € | 4.17 | 247 € | -1.88 | -131 | 32.1 | 1.3 | 0.6 | 66.0 | - | - | - |  |
| Counselling | 27 | 217 € | 2.30 | 1 € | -0.01 | -171 | 18.7 | 28.5 | 20.5 | 32.3 | - | - | - |  |

**eTable 2. Mean total costs and unadjusted QALY per person at 1-year follow-up (6-month intervention and 6-month follow-up) and incremental cost-effectiveness ratio (ICER)**

|  | | | | **Compared with control group** | | | | | | | | | | |
| --- | --- | --- | --- | --- | --- | --- | --- | --- | --- | --- | --- | --- | --- | --- |
| **Intervention groups** | **Sample size** | **Cost / person (C)** | **QALY / person (E)** | **Incremental cost (ΔC)** | **Incremental effect (ΔE)** | **ICER**  **(ΔC / ΔE)** | **NE** | **SE** | **SW** | **NW** | **85% ICER** | **90% ICER** | **95% ICER** |  |
| Control | 34 | 1,088 € | 0.30 |  |  |  |  |  |  |  |  |  |  |  |
| Combination | 33 | 480 € | 0.33 | -608 € | 0.03 | -18,699 | 8.7 | 42.7 | 40.7 | 7.9 | - | - | - |  |
| Pilates | 30 | 844 € | 0.32 | -244 € | 0.03 | -9,763 | 18.9 | 31.8 | 28.7 | 20.6 | - | - | - |  |
| Counselling | 24 | 1,202 € | 0.44 | 114 € | 0.15 | 788 | 35.1 | 19.3 | 20.5 | 25.1 | - | - | - |  |


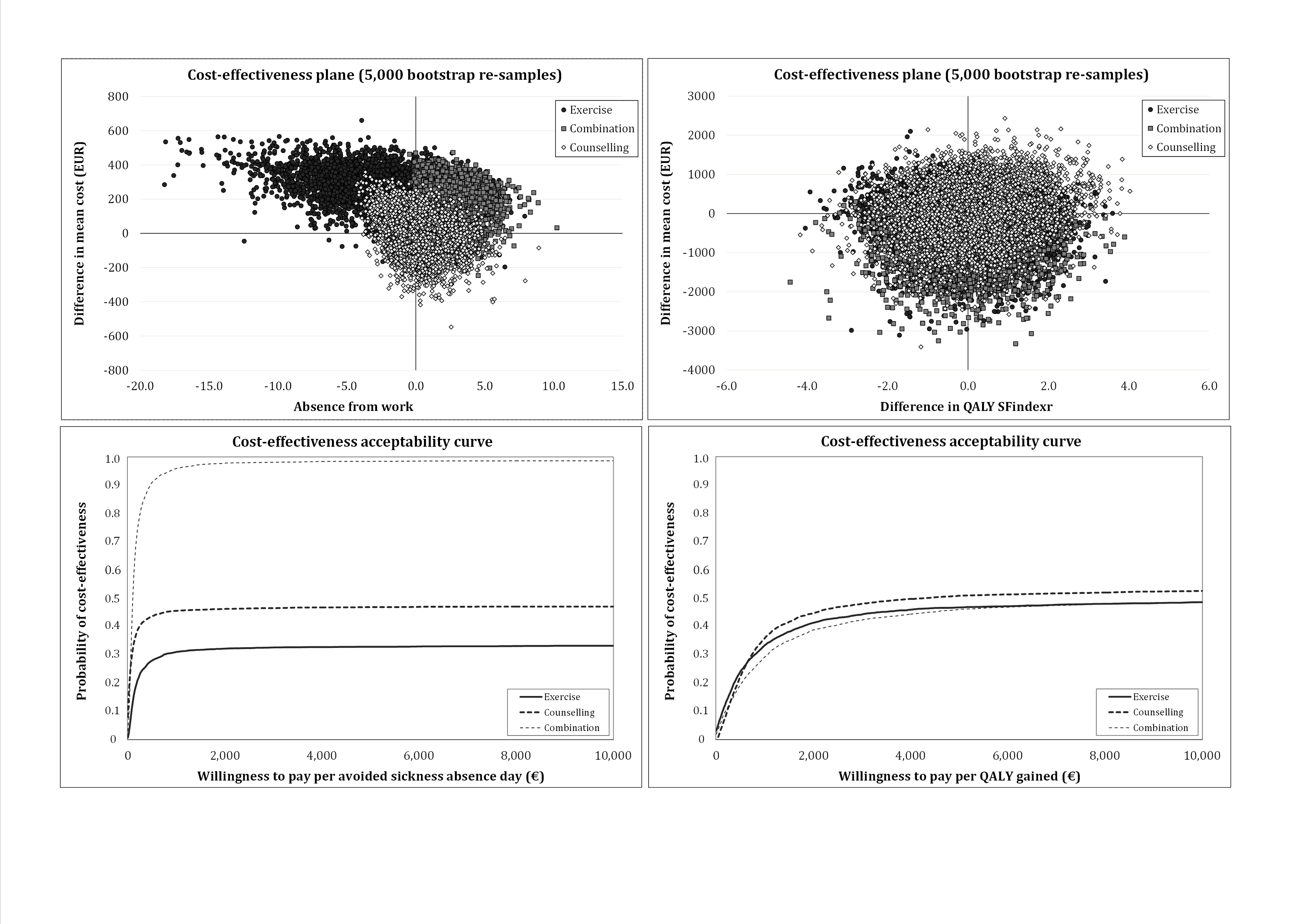


**eFigure1. Unadjusted cost-effectiveness plane and acceptability curves for absence from work and for QALY**

**e2.2. Adjusted cost-effectiveness/utility outcomes (adjusted for baseline differences with the reported absence days and QALYs): Tables e3-e4:**

**eTable 3. Mean total costs and adjusted absence from work per person at 1-year follow-up (6-month intervention and 6-month follow-up) and incremental cost-effectiveness ratio (ICER)**

|  | | | | **Compared with control group** | | | | | | | | | |
| --- | --- | --- | --- | --- | --- | --- | --- | --- | --- | --- | --- | --- | --- |
| **Intervention groups** | **Sample size** | **Cost / person (C)** | **Absence from work/ person (E) *** | **Incremental cost (ΔC)** | **Incremental effect (ΔE) *** | **ICER**  **(ΔC / ΔE)** | **NE** | **SE** | **SW** | **NW** | **85% ICER** | **90% ICER** | **95% ICER** |
| Control | 37 | 222 € | 1.92 |  |  |  |  |  |  |  |  |  |  |
| Combination | 34 | 421 € | 0.36 | 199 € | 2.12 | 93.9 | 95.1 | 3.2 | 0.1 | 1.6 | 407 € | 582 € | 1,059 € |
| Exercise | 35 | 463 € | 4.42 | 241 € | -2.50 | -96.7 | 30.3 | 1.4 | 0.3 | 67.0 | - | - | - |
| Counselling | 27 | 217 € | 2.29 | -5 € | -0.02 | 293.6 | 20.0 | 28.0 | 21.2 | 30.8 | - | - | - |

**eTable 4. Mean total costs and adjusted QALY per person at 1-year follow-up (6-month intervention and 6-month follow-up) and incremental cost-effectiveness ratio (ICER)**

|  | | | | **Compared with control group** | | | | | | | | | |
| --- | --- | --- | --- | --- | --- | --- | --- | --- | --- | --- | --- | --- | --- |
| **Intervention groups** | **Sample size** | **Cost / person (C)** | **QALY / person (E)** | **Incremental cost (ΔC)** | **Incremental effect (ΔE)** | **ICER**  **(ΔC / ΔE)** | **NE** | **SE** | **SW** | **NW** | **85% ICER** | **90% ICER** | **95% ICER** |
| Control | 34 | 1,088 € | 0.13 |  |  |  |  |  |  |  |  |  |  |
| Combination | 33 | 480 € | -0.35 | -608 € | -0.43 | 1,403 | 4.4 | 27.6 | 55.8 | 12.2 | - | - | - |
| Exercise | 30 | 844 € | 1.05 | -244 € | 1.00 | -240 | 32.0 | 54.6 | 5.9 | 7.6 | 3,550 € | - | - |
| Counselling | 24 | 1,202 € | 0.71 | 114 € | 0.71 | 162 | 47.2 | 31.1 | 8.6 | 13.1 | - | - | - |

**eReferences**

[1] Kolu P, Tokola K, Kankaanpää M, Suni J. Evaluation of the effects of physical activity, cardiorespiratory condition, and neuromuscular fitness on direct health-care costs and sickness-related absence among nursing personnel with recurrent nonspecific low back pain. *Spine* (Phila Pa 1976) 2017;42:854−62.

[2] Aalto A-M, Aro, A R , Teperi, J. *RAND-36 as a measure of Health-Related Quality of Life. Reliability, construct validity and reference values in the Finnish general population.* RAND-36 terveyteen liittyvän elämänlaadun mittarina. Mittarin luotettavuus ja suomalaiset väestöarvot. Helsinki: Stakes, Tutkimuksia 101, 1999,9:49−51.

[3] Ware JE, Jr, Sherbourne CD. The MOS 36-item short-form health survey (SF-36) I. Conceptual framework and item selection. *Med Care* 1992;30:473−83.

[4] Brazier J, Roberts J, Deverill M. The estimation of a preference-based measure of health from the SF-36*. J Health Econ* 2002;21:271−92.

[5] McCabe C, Brazier J, Gilks P, et al. Using rank data to estimate health state utility models. *J Health Econ* 2006;25:418−31.

[6] Kharroubi SA, Brazier JE, Roberts J, O'Hagan A. Modelling SF-6D health state preference data using a nonparametric Bayesian method. *J Health Econ* 2007;26:597−612.

[7] Kapiainen S, A V, Haula T. Health-care Unit Costs in Finland, 2011 (available in Finnish); doi Helsinki, Finland: National Institute for Health and Welfare; 2014.

[8] Price index of public expenditure [e-publication]. Statistics Finland, 2015a. (Accessed 22/11/2016; doi: at http://www.stat.fi/til/jmhi/index_en.html.)

[9] Wage structure [e-publication, in Finnish] Statistics Finland, 2015b. (Accessed 22/11/2016; doi: at <http://pxnet2.stat.fi/PXWeb/pxweb/fi/StatFin/StatFin__pal__pra__2013/?tablelist=true>.
